# Longitudinal Maternal Vitamin D Status during Pregnancy Is Associated with Neonatal Anthropometric Measures

**DOI:** 10.3390/nu10111631

**Published:** 2018-11-02

**Authors:** Ellen C. Francis, Stefanie N. Hinkle, Yiqing Song, Shristi Rawal, Sarah R. Donnelly, Yeyi Zhu, Liwei Chen, Cuilin Zhang

**Affiliations:** 1 Epidemiology Branch, Division of Intramural Population Health Research, Eunice Kennedy Shriver National Institute of Child Health and Human Development, National Institutes of Health, Bethesda, MD 20892, USA; ellen.francis@NIH.gov (E.C.F.); stefanie.hinkle@nih.gov (S.N.H.); shristi.rawal@rutgers.edu (S.R.); srdonnelly04@vt.edu (S.R.D.); 2 Department of Public Health Sciences, Clemson University, Clemson, SC 29634, USA; ecfranc@clemson.edu or liweic@clemson.edu; 3 Department of Epidemiology, Indiana University Richard M. Fairbanks School of Public Health, Indianapolis, IN 47405, USA; yiqsong@iu.edu; 4 Department of Nutritional Sciences, Rutgers School of Health Professions, Newark, NJ 07102, USA; 5 Department of Human Nutrition, Foods, and Exercise, Virginia Tech, Blacksburg VA 24061, USA; 6 Division of Research, Kaiser Permanente Northern California, Oakland, CA 94612 USA; yeyi.zhu@kp.org

**Keywords:** vitamin D, neonate anthropometry, fetal growth, maternal, infant

## Abstract

Findings on maternal 25-hydroxyvitamin D (25[OH]D) and neonatal anthropometry are inconsistent, and may at least be partly due to variations in gestational week (GW) of 25(OH)D measurement and the lack of longitudinal 25(OH)D measurements across gestation. The aim of the current study was to examine the associations of longitudinal measures of maternal 25(OH)D and neonatal anthropometry at birth. This study included 321 mother–offspring pairs enrolled in the *Eunice Kennedy Shriver* National Institute of Child Health and Human Development Fetal Growth Studies–Singletons. This study was a prospective cohort design without supplementation and without data on dietary supplementation. Nevertheless, measurement of plasma 25(OH)D reflects vitamin D from different sources, including supplementation. Maternal concentrations of total 25(OH)D were measured at 10–14, 15–26, 23–31, and 33–39 GW and categorized as <50 nmol/L, 50–75 nmol/L, and >75 nmol/L. Generalized linear models were used to examine associations of 25(OH)D at each time-point with neonate birthweight z-score, length, and sum of skinfolds at birth. At 10–14 GW, 16.8% and 49.2% of women had 25(OH)D <50 nmol/L and between 50–75 nmol/L, respectively. The association of maternal 25(OH)D with neonatal anthropometry differed by GW and women’s prepregnancy BMI (normal (<25.0 kg/m^2^), overweight/obese (25.0–44.9 kg/m^2^)). All analyses were stratified by prepregnancy BMI status. Among women with an overweight/obese BMI, 25(OH)D <50 nmol/L at 10–14 GW was associated with lower birthweight z-score (0.56; 95% CI: −0.99, −0.13) and length (−1.56 cm; 95% CI: −3.07, −0.06), and at 23–31 GW was associated with shorter length (−2.77 cm; 95% CI: −13.38, −4.98) and lower sum of skinfolds (−9.18 mm; 95% CI: −13.38, −4.98). Among women with a normal BMI, 25(OH)D <50 nmol/L at 10–14 GW was associated with lower sum of skinfolds (−2.64 mm; 95% CI: −5.03, −0.24), at 23–31 GW was associated with larger birthweight z-scores (0.64; 95% CI: 0.03, 1.25), and at 33-39 GW with both higher birthweight z-score (1.22; 95% CI: 0.71, 1.73) and longer length (1.94 cm; 95% CI: 0.37, 3.52). Maternal 25(OH)D status during pregnancy was associated with neonatal anthropometric measures, and the associations were specific to GW of 25(OH)D measurement and prepregnancy BMI.

## 1. Introduction

Although the classical function of vitamin D is to regulate calcium and phosphorus metabolism in the intestine and bone, recent findings indicate its important role in several other biochemical and physiological processes, including regulation of the immune system, cellular differentiation, and blood pressure [1]. In humans, 25(OH)D, the hydrolyzed form of vitamin D, is the predominant form of circulating vitamin D and is considered the clinical standard for measuring bioactive vitamin D status [2]. Maternal 25(OH)D levels during pregnancy have been considered critical for both maternal health and fetal development [2,3,4,5,6]. Lower maternal 25(OH)D levels have been associated with unfavorable fetal growth outcomes, such as low birth weight, shorter bone length, and small-for-gestational age (SGA) births in some, though not all studies [7,8,9,10]. The inconsistent results in the literature may be partially caused by differences in timing of 25(OH)D measurement; for example, some studies have measured maternal 25(OH)D concentration early in pregnancy at 11–13 gestational weeks (GW) [7], while others have been later in pregnancy at 28–32 GW [10].

Rapid cardiometabolic and hormonal changes during pregnancy results in dynamic alterations in maternal 25(OH)D metabolism and circulating concentrations throughout pregnancy [6]. There is some evidence from recent studies that 25(OH)D increases throughout pregnancy [11,12,13,14]. As such, the gestational age when maternal 25(OH)D is measured may play a role in different findings of the associations between 25(OH)D and neonatal anthropometry. To our knowledge, only one study has examined maternal 25(OH)D measured twice during pregnancy (before 16 GW and 24–28 GW) and only maternal 25(OH)D concentrations at 24–28 GW were inversely associated with newborn knee–heel length [9]. The lack of longitudinal data on maternal 25(OH)D status at multiple time-points throughout pregnancy has limited our understanding of the association between maternal vitamin D status, particularly at specific developmental windows, and fetal growth [15,16]. Therefore, the current study aimed to examine the longitudinal associations of maternal 25(OH)D concentrations at multiple time-points throughout pregnancy and neonatal anthropometry, including birthweight, length, and sum of skinfolds at birth.

## 2. Materials and Methods

### 2.1. Study Population

The current study was based on data from a nested case-control study within the *Eunice Kennedy Shriver* National Institute of Child Health and Human Development (NICHD) Fetal Growth Studies–Singletons (2009–2013) [17]. Between 8–13 GWs, low-risk pregnant women without a history of chronic or medical condistions (e.g., prepregnancy hypertension, autoimmune disorders) were enrolled and followed through delivery. Extensive details on study design and participant characteristics have been previously published [17]. Women were recruited from 12 clinics across the US: Columbia University (NY), New York Hospital, Queens (NY), Christiana Care Health System (DE), Saint Peter’s University Hospital (NJ), Medical University of South Carolina (SC), University of Alabama (AL), Northwestern University (IL), Long Beach Memorial Medical Center (CA), University of California, Irvine (CA), Fountain Valley Hospital (CA), Women and Infants Hospital of Rhode Island (RI), and Tufts University (MA). Written consent was obtained from all participants and institutional Review Board approval was obtained for all participating clinical sites, the data coordinating center, and NICHD (09-CH-N152). This study was carried out following the rules of the Declaration of Helsinki. The current study included 321 mother–offspring pairs who had maternal vitamin D biomarkers measured throughout pregnancy. This nested case-control study comprised women with gestational diabetes mellitus (GDM) (*n* = 107) and controls (*n* = 214) matched at a ratio of 1:2 on maternal age (±2 years), race/ethnicity, and GW (±2 weeks) at blood collection.

### 2.2. Assessment of Maternal Vitamin D

As a planned component of the NICHD Fetal Growth Studies-Singletons, maternal biospecimens were collected four times during pregnancy (10–14, 15–26, 23–31, and 33–39 GW) [17]. Maternal plasma vitamin D biomarkers were measured for all GDM cases and controls at 10–14 and 15–26 GW. At 23–31 and 33–39 GWs, one of the two controls was randomly selected and biomarkers were assayed in this same control at the later time-points. Within the larger prospective cohort study, and within the nested case-control, no vitamin D supplementation was provided for study purposes. Plasma concentrations of 25(OH)D2 and 25(OH)D3 (ng/mL) were measured using liquid chromatography–mass spectrometry (LC–MS). Total 25(OH)D was calculated as the sum of 25(OH)D2 and 25(OH)D3 and reported in nmol/L using the conversion unit of 2.5 [18].

### 2.3. Assessment of Neonatal Anthropometric Measurements

Gestational age- and sex-specific birthweight z-scores were derived using birthweight abstracted from medical records [19]. Neonatal anthropometric measures were collected after delivery (median 1 day; interquartile range 1–2 days). Measurements were obtained in at least duplicate using standard protocol [20,21,22]. Neonatal crown–heel length (cm) was measured using an infantometer, and skinfold thickness (mm) was measured using a Lange skinfold caliper. Abdominal flank, anterior thigh, subscapular, and tricep skinfolds were summed (sum of skinfolds) as a measure of neonatal adiposity. One of the clinical sites used the incorrect calipers and, thus, participants from this site were excluded from skinfold analyses (*n* = 12).

### 2.4. Covariates

Maternal sociodemographic characteristics were collected from detailed questionnaires at enrollment. At enrollment (8–13 GW), prepregnancy body mass index kg/m^2^ (BMI) was calculated based on self-reported weight and measured height. Self-reported weight was highly correlated with weight subsequently measured by study personnel during the enrollment visit (correlation coefficients of 0.97) [23]. Prepregnancy BMI was categorized as normal weight (<25.0), overweight (25.0–29.9), or obese (≥30.0). Physical activity (PA) was assessed at enrollment regarding habitual PA, and at subsequent study visits regarding PA since the prior visit [24]. Clinical centers were grouped into three categories based on latitude (southern ≤37° N; middle 38° N–40° N; northern >40° N latitude) [25]. Season of blood collection was categorized as winter (January–March), spring (April–June), summer (July–September), and fall (October–December).

### 2.5. Statistical Analysis

Sampling weights were applied to all analyses to represent the full NICHD Fetal Growth Studies–Singletons population and account for the oversampling of women with GDM in the case-control study [26,27]. Following visual inspection of the data and residuals, normality of distribution was confirmed and therefore parametric models were fitted to the data. Descriptive statistics were presented as weighted mean ± standard error (SE) for continuous variables, and frequency and weighted percent for categorical variables. Significant differences among descriptive statistics were based on *t*-test for continuous variables and chi-square for categorical variables, with both standard errors and *P*-values for differences based on robust variance estimates. Generalized linear models with robust SE were used to examine associations between maternal total 25(OH)D at each visit and neonatal anthropometric measures of birthweight z-score, length, and sum of skinfolds. A test for a linear trend across quartiles was performed by fitting the median value for each quartile as a continuous variable in generalized linear models. Additionally, restricted cubic splines were used to test for nonlinear associations between maternal 25(OH)D and neonatal anthropometry, but a nonlinear relationship was not found.

Maternal 25(OH)D was examined both continuously and categorically. Categories of 25(OH)D were examined based on the distribution at each visit (quartiles), and based on cutoffs of <50 nmol/L, 50–75 nmol/L, and >75 nmol/L [1]. Currently, there is no consensus for 25(OH)D deficiency specific to pregnancy; thus, commonly used cutoffs when assessing 25(OH)D in pregnant women were used [8,28] and that would result in an adequate sample size in each category based on the distribution of 25(OH)D in our sample. Neonatal anthropometric outcomes were treated as continuous variables.

All models were adjusted for maternal matching factors, including maternal age (continuous), race/ethnicity (non-Hispanic White, non-Hispanic Black, Hispanic, Asian and Pacific Islander), and GW at blood draw. Additional covariates included education (high-school degree or less, Associate degree, Bachelor degree), prepregnancy BMI (continuous), marital status (married/living with a partner or not), and insurance (private/managed care or Medicaid/other). Models of neonatal length and sum of skinfolds were further adjusted for the number of days between delivery and measurement date.

Several sensitivity analyses were conducted to test the robustness of findings. Analyses were stratified by offspring gender, prepregnancy BMI (normal versus overweight/obese), race/ethnicity (non-Hispanic Black versus not), and PA at enrollment (<median versus ≥ median level). To examine the change in 25(OH)D status across pregnancy, three profiles of 25(OH)D concentrations throughout pregnancy were determined: (1) Consistently <50 nmol/L, (2) an alternating status ranging across all concentration categories, and (3) consistently >75 nmol/L. In addition, controlling for gestational weight gain by taking the difference between the weight at each time-point and the woman’s prepregnancy weight was explored. All analyses were implemented using SAS Version 9.4 (SAS Institute, Cary, NC, USA), with α < 0.05 as the level of significance.

## 3. Results

The mean ± SE levels of maternal 25(OH)D were 68.9 (1.5) nmol/L at 10–14 GW, 76.2 (1.8) nmol/L at 15–26 GW, 80.9 (2.7) nmol/L at 23–31 GW, and 82.5 (3.1) nmol/L at 33–39 GW. The percentage of women with 25(OH)D <50 nmol/L changed throughout pregnancy, with 16.8% at 10–14 GW, 11.1% at 15–26 GW, 11.2% at 23–31 GW, and 8.3% at 33–39 GW. The mean ± SE birthweight z-score was 0.22 (0.07), neonatal length was 50.3 (0.23) cm, and sum of skinfolds was 19.7 (0.4) mm. At enrollment (10–14 GW), maternal 25(OH)D was associated with race/ethnicity, prepregnancy BMI, education, insurance type, marital status, and PA, but not with maternal age, parity, smoking, season, or clinic location (Table 1).

Associations of maternal 25(OH)D and neonatal anthropometry varied by maternal prepregnancy BMI status (Table 2 and Table 3). There were no substantial differences from our main results when controlling for gestational weight gain, when examining profiles of 25(OH)D throughout gestation (Figure S1), or in stratified analyses by offspring gender, maternal race/ethnicity, PA level at enrollment, or GDM status. In the following sections, the results of maternal 25(OH)D levels categorized as <50 nmol/L, 50–70 nmol/L, and >75 nmol/L and stratified by maternal prepregnancy BMI status are presented. Results of maternal 25(OH)D levels categorized by quartiles in relation to neonatal anthropometry can be found in Table S1, unstratified results of 25(OH)D levels categorized as <50 nmol/L, 50–70 nmol/L, and >75 nmol/L can be found in Table S2, and frequencies of women with GDM in each 25(OH)D category can be found in Table S3.

### 3.1. Total 25(OH)D and Neonatal Birthweight Z-Score, Length, and Sum of Skinfold in Women with Prepregnancy Overweight/Obese BMI (>25 kg/m^2^)

Among women with prepregnancy overweight/obesity, maternal 25(OH)D was negatively associated with offspring birthweight z-score, length, and sum of skinfolds, but the strength of the association varied by exposure window during pregnancy (Table 2). At 10–14 GW, neonates of women with 25(OH)D <50 nmol/L had a lower birthweight (*p* = 0.01) and shorter length (*p* = 0.04) than neonates of women with 25(OH)D >75 nmol/L. At 23–31 GW, neonates of women with 25(OH)D <50 nmol/L had shorter length (*p* = 0.001) and lower sum of skinfolds (*p* <0.0001) than neonates of women with 25(OH)D >75 nmol/L. At 33–39 GW, neonates of women with 25(OH)D <50 nmol/L had shorter length (*p* = 0.02) than neonates of women with 25(OH)D >75 nmol/L.

### 3.2. Total 25(OH)D and Neonatal Birthweight Z-Score, Length, and Sum of Skinfold in Women with Prepregnancy Normal BMI (18.5–24.9 kg/m^2^)

Among women with a normal prepregnancy BMI, the direction of associations of maternal 25(OH)D with neonatal anthropometry varied by exposure window during pregnancy (Table 3). At 10–14 GW, neonates of women who had 25(OH)D <50 nmol/L had lower sum of skinfolds (*p* = 0.03) than neonates of women with 25(OH)D >75 nmol/L; similar findings were observed for 25(OH)D concentrations between 50–75 nmol/L. At 23–31 GW, neonates of women with 25(OH)D <50 nmol/L had larger birthweight z-scores (*p* = 0.04) than neonates of women with 25(OH)D >75 nmol/L. At 33–39 GW, neonates of women with 25(OH)D <50 nmol/L had larger birthweight z-scores (*p* <0.0001) and (*p* = 0.02) larger length compared to neonates of women with 25(OH)D >75 nmol/L.

## 4. Discussion

In the current study, the direction of association between maternal 25(OH)D and neonatal anthropometry varied by maternal prepregnancy adiposity status and GW of 25(OH)D measurement. Although maternal 25(OH)D is recognized to play an important role in fetal growth, due to a lack of studies with longitudinal measures of 25(OH)D during pregnancy in relation to neonatal anthropometry, direct comparison of our results with previous findings is challenging. In the following sections, the congruency of our results based on the time-point during pregnancy when 25(OH)D was measured and neonatal outcome is discussed.

### 4.1. Maternal 25(OH)D and Neonatal Birthweight

Most studies reporting a positive association between maternal 25(OH)D and neonatal birthweight mostly included women with prepregnancy normal weight, limiting the comparability to our study [29,30]. A study that measured maternal 25(OH)D multiple times during pregnancy (11–16 and 28–32 GW) found no associations between 25(OH)D <28 nmol/L at either time-point and neonatal birthweight [9]. Although they controlled for maternal BMI, the mean BMI was not reported. Several other studies were based on a single time-point and used much lower cutoffs for 25(OH)D (i.e., <25 nmol/L), and reported a positive association between maternal 25(OH)D in later pregnancy and birthweight [31,32]. We are not aware of any observational studies that observed a negative association between maternal 25(OH)D after 32 GW and birthweight and thus our findings among women with normal weight require replication.

### 4.2. Maternal 25(OH)D and Neonatal Length

A previous study, which examined maternal 25(OH)D twice during pregnancy, found no association of 25(OH)D at 11–16 GW, but, similar to our results, found a positive association at 28–32 GW with neonatal length [9]. That study did not stratify by BMI or report the mean BMI and, thus, direct comparisons with our study are challenging. To our knowledge, no study has examined associations of maternal 25(OH)D in late pregnancy (>32 GW) and neonatal length. The finding of significant associations in late pregnancy (33–39 GW), regardless of prepregnancy BMI status, has not been previously reported and warrants confirmation.

### 4.3. Maternal 25(OH)D and Neonatal Sum of Skinfolds

Previously, a positive association between 25(OH)D measured at 28–32 GW and neonatal subscapular skinfold thickness was observed [9], but again, the adiposity status of the women was not reported. Contrary to our findings, a study among mostly women with normal weight found no association between maternal 25(OH)D at <26 GW and neonatal adiposity as measured by ponderal index [30].

Differences among study findings may be related to study design, population, timing when the maternal 25(OH)D was measured, and distribution of 25(OH)D concentrations. In some studies, there was a high proportion of women with extremely low concentrations of 25(OH)D [29,31,32,33], whereas in our study, less than 20% of women had 25(OH)D <50 nmol/L at any time during pregnancy. The increase in 25(OH)D throughout gestation is typical of the pregnant state and is in response to the physiological demands of pregnancy [2].

The exposure window during pregnancy when maternal 25(OH)D may impact neonatal size is important to consider in relation to fetal growth in utero. In early pregnancy, bones and muscles begin to grow, including the formation of arms, legs, backbone, and neck [34]. In late pregnancy, the fetus gains weight mainly through accumulation of fat mass and bone density [34]. Although the association of 25(OH)D and neonate anthropometry at birth was dependent on GW, there are many factors that contribute to fetal growth. It is likely the interplay of vitamin D with many other hormones and nutrients that results in the overall body composition of the neonate at birth. For instance, maternal calcium absorption and placental calcium transfer both increase to meet fetal demands and are responsive to 1,25(OH)_2_D, the biologically active form of vitamin D [35]. Calcium serves as a key structural component in bone development, with higher concentrations needed for the fetus to effectively mineralize the skeleton [36]. The role of vitamin D in calcium absorption may therefore also impact fetal skeletal muscle and bone development. The concentration of calcium available to the fetus is heavily dependent on maternal concentrations, the latter of which has been reported to explain 3% of the variance in birth length [37]. Therefore, maternal vitamin D status, as reflected by 25(OH)D concentrations, may represent its role in skeletal function in fetal growth. On the other hand, adequate maternal vitamin D status has been favorably associated with improving maternal glucose and insulin homeostasis [38], which may have a downstream impact on the glucose load experienced by the fetus, which in turn may curb excessive fetal growth in late gestation. In addition, vitamin D’s role in immune function, systemic inflammation, and endothelial function is important for normal placental function. Maternal concentrations of 25(OH)D may also stimulate secretion of placental hormones that facilitate fetal growth, such as placental lactogen [6].

Inverse associations of maternal 25(OH)D with birthweight and length among women with prepregnancy normal weight has not been reported before. In our study, women with prepregnancy overweight/obesity had lower 25(OH)D concentrations at enrollment than women with normal weight. The differences in synthesis and metabolism of vitamin D among individuals with and without obesity is still under investigation [39,40], and the impact of these differences on fetal growth is unclear.

### 4.4. Strengths and Limitations

The current study has several strengths, including prospective longitudinal data collection, thereby allowing the investigation of gestation-specific associations of maternal 25(OH)D and neonatal anthropometry. Data on plasma concentrations of 25(OH)D were used, which is downstream of supplement and dietary sources and has been reported to be the most accurate indicator of total exposure to vitamin D from all sources [3]. Moreover, study participants were enrolled from geographically diverse US clinics and represented various race/ethnicities. Detailed data on potential confounders during and prior to pregnancy were available and controlled for when appropriate, and interactions with offspring gender, race/ethnicity, and maternal prepregnancy BMI status were explored. Although we have controlled for known major confounders, similar to other observational studies, we cannot completely exclude the possibility for residual confounding by unmeasured factors or measurement errors. In the current study, there was a relatively small sample size, which precluded us from examining extreme phenotypes of fetal growth, such as small- or large-for-gestational age. Lastly, self-reported prepregnancy weight was used to calculate prepregnancy BMI upon recruitment into the cohort. However, self-reported weight was highly correlated with measured maternal weight (r = 0.97) in this population and other studies [23,41].

### 4.5. Suggestions for Future Research

In addition to investigating maternal 25(OH)D status during different time windows of pregnancy in association with neonatal anthropometry, our study further evaluated whether the impact of maternal vitamin D status on offspring anthropometric measures varied by maternal prepregnancy BMI (i.e., normal weight vs. overweight/obese), which has not been previously investigated in the literature. In the current study, there was a difference in direction of association between 25(OH)D and neonatal anthropometry by BMI categories (i.e., at 33–39 GW, there was an inverse association with length among women with a prepregnancy BMI in the normal range, but a positive association among women with a prepregnancy BMI in the overweight/obese range). Future investigations are warranted to replicate these findings. If confirmed, these findings indicate that endeavors to optimize maternal 25(OH)D status should likely consider women’s prepregnancy adiposity status and the specific neonatal anthropometric outcome, both of which are justifications for efforts into precision nutrition.

## 5. Conclusions

If confirmed, our findings highlight the significance of the concept of precision nutrition, which considers tailored approaches to 25(OH)D supplementation to improve fetal outcomes by considering timing of GW and maternal adiposity status. At least among women who were overweight/obese before pregnancy, low 25(OH)D (<50 nmol/L) in both early and late pregnancy may impact fetal development. Considering that almost half of US women entering pregnancy are overweight or obese, prevention of low 25(OH)D concentrations in early and late pregnancy may be particularly relevant to optimizing fetal growth.

## Figures and Tables

**Table 1:** Characteristics and maternal 25(OH)D nmol/L concentrations at enrollment ^1^.

Characteristics	*N* (%)	Total 25(OH) DMean ± SE	*p*
All	321	68.9 (1.5)	-
Age (mean age, years) ^2^	28.2 ± 0.5	-	-
<25	54 (30.0)	65.1 ± 3.0	
25–29	85 (29.7)	70.6 ± 4.3	
30–34	97 (25.8)	67.4 ± 4.0	
≥35	85 (14.5)	76.1 ± 4.6	0.11
Race/ethnicity			
Non-Hispanic white	75 (30.9)	81.3 ± 2.7	
Non-Hispanic black	45 (23.3)	58.3 ± 4.2	
Hispanic	123 (27.2)	66.8 ± 3.4	
Asian/Pacific Islander	78 (18.5)	64.6 ± 4.1	<0.001
Prepregnancy BMI (mean, kg/m^2^) ^2^	25.8 ± 0.4	-	-
Normal	162 (56.1)	72.3 ± 2.1	
Overweight	91 (28.7)	61.6 ± 3.3	
Obese	68 (15.1)	66.1 ± 3.6	0.01
Education (degree)			
High school or less	81 (25.1)	66.0 ± 2.4	
Associates	117 (35.2)	64.6 ± 3.5	
Bachelor’s or higher	123 (39.8)	74.6 ± 3.6	0.02
Insurance			
Private or managed care	211 (64.6)	71.1 ± 3.0	
Medicaid, other	108 (35.4)	64.9 ± 2.2	0.04
Marital Status			
Not married	62 (27.1)	62.9 ± 3.0	
Married/living with a partner	259 (72.9)	71.2 ± 3.5	0.02
Nulliparous			
Yes	144 (51.1)	69.3 ± 3.1	
No	177 (48.9)	68.4 ± 2.2	0.77
Physical activity MET score Type-Sports/exercise ^2^	11.1 ± 0.78	-	-
≥50th percentile	165 (50.3)	73.8 ± 3.0	
<50th percentile	156 (49.7)	64.0 ± 2.1	0.002
Smoking 6 months prepregnancy			
Yes	5 (0.7)	73.4 ± 4.3	
No	316 (99.3)	68.9 ± 4.6	0.54 ^3^
Season of study enrollment			
Winter	89 (33.3)	67.8 ± 2.7	
Fall	71 (20.1)	67.4 ± 4.2	
Spring	78 (21.2)	69.3 ± 4.2	
Summer	82 (25.4)	72.1 ± 4.2	0.72
Clinic Location			
Southern (≤37° N)	117 (37.7)	67.2 ± 2.6	
Middle (38° N–40° N)	145 (44.4)	67.7 ± 4.4	
Northern (>40° N)	59 (17.9)	70.8 ± 3.4	0.54

^1^ Participant characteristics are presented as frequency (weighted percent), total 25(OH)D (nmol/L) are presented as mean ± SE. ^2^ Represents mean ± SE for a continuous variable. ^3^ *p-*value not based on robust variance estimates due to small cell size.

**Table 2:** Longitudinal associations of maternal total 25(OH)D (nmol/L) and birthweight z-score, length (cm), and sum of skinfolds (mm) among women with an overweight/obese prepregnancy BMI ^1^.

Maternal 25(OH)D Status	*n*	10–14 GW	*n*	15–26 GW	*n*	23–31 GW	*n*	33–39 GW
Birthweight z-score								
<50 nmol/L	45	−0.56 (−0.99, −0.13) *	24	0.15 (−0.51, 0.80)	11	−0.52 (−1.00, −0.04) *	12	−0.17 (−0.74, 0.40)
50–75 nmol/L	73	−0.35 (−0.68, −0.03) *	73	0.04 (−0.33, 0.41)	46	−0.13 (−0.58, 0.32)	43	0.29 (−0.17, 0.76)
>75 nmol/L	32	Reference	51	Reference	52	Reference	46	Reference
Length								
<50 nmol/L	27	−1.56 (−3.07, −0.06) *	22	0.98 (−1.01, 2.98)	11	−2.77 (−4.43, −1.12) *	11	−1.98 (−3.66, −0.31) *
50–75 nmol/L	6 8	−2.04 (−3.37, −0.71) *	64	1.06 (−0.61, 2.74)	43	−2.40 (−3.71, −1.08) *	38	1.76 (0.32, 3.19) *
>75 nmol/L	41	Reference	49	Reference	49	Reference	44	Reference
Sum of Skinfolds								
<50 nmol/L	25	−0.52 (−4.34, 3.30)	18	3.57 (−0.43, 7.57)	9	−9.18 (−13.38, −4.98) *	9	−4.29 (−8.75, 0.17)
50–75 nmol/L	63	0.69 (−2.30, 3.69)	63	4.94 (2.36, 7.52) *	43	−0.85 (−3.86, 2.17)	39	2.52 (−1.42, 6.46)
>75 nmol/L	37	Reference	44	Reference	46	Reference	40	Reference

^1^ Data are presented as regression coefficients and confidence intervals (CI) and reflect the differences in neonatal anthropometry compared to the reference group (25(OH)D >75 nom/L). All models are adjusted for maternal matching characteristics (age (continuous), race, and gestational age at blood collection), and adjusted for education, insurance type, marital status, and prepregnancy BMI (continuous). Models of sum of skinfolds were adjusted to account for the difference in days between birth and date of anthropometric measurement. * (*p*-value < 0.005).

**Table 3:** Longitudinal associations of maternal total 25(OH)D (nmol/L) and birthweight z-score, length (cm), and sum of skinfolds (mm) among women with a normal prepregnancy BMI ^1^.

Maternal 25(OH)D Status	*n*	10–14 GW	*n*	15–26 GW	*n*	23–31 GW	*n*	33–39 GW
Birthweight z-score								
<50 nmol/L	24	0.05 (−0.40, 0.51)	12	0.09 (−0.49, 0.67)	6	0.64 0.03, 1.25) *	3	1.22 (0.71, 1.73) *
50–75 nmol/L	65	−0.15 (−0.50, 0.20)	48	−0.26 (−0.59, 0.07)	22	0.08 (−0.49, 0.65)	26	−0.01 (−0.37, 0.34)
>75 nmol/L	67	Reference	94	Reference	64	Reference	57	Reference
Length								
<50 nmol/L	23	0.67 (−0.93, 2.28)	12	0.54 (−1.54, 2.62)	6	1.61 (−0.34, 3.55)	3	1.94 (0.37, 3.52) *
50–75 nmol/L	59	−0.24 (−1.32, 0.85)	44	−0.34 (−1.19, 0.52)	22	−0.47 (−1.59, 0.64)	26	0.19 (−0.89, 1.28)
>75 nmol/L	62	Reference	87	Reference	59	Reference	52	Reference
Sum of Skinfolds								
<50 nmol/L	19	−2.64 (−5.03, −0.24) *	11	−0.15 (−2.34, 2.05)	6	0.84 (−2.52, 4.21)	3	1.29 (−2.22, 4.80)
50–75 nmol/L	59	−2.32 (−4.00, −0.63) *	42	−1.42 (−3.04, 0.19)	20	−0.66 (−3.12, 1.80)	24	1.83 (−0.37, 4.03)
>75 nmol/L	60	Reference	84	Reference	58	Reference	53	Reference

^1^ Data are presented as regression coefficients and confidence intervals (CI) and reflect the differences in neonatal anthropometry compared to the reference group (25(OH)D >75 nom/L). All models are adjusted for maternal matching characteristics (age (continuous), race, and gestational age at blood collection), and adjusted for education, insurance type, marital status, and prepregnancy BMI (continuous). Models of sum of skinfolds were adjusted to account for the difference in days between birth and date of anthropometric measurement. * (*p*-value < 0.005).

## Supplementary Materials

The following are available online at http://www.mdpi.com/2072-6643/10/11/1631/s1, Table S1: Longitudinal associations of maternal total 25(OH)D and neonatal birthweight z-score, length (cm), and sum of skinfolds stratified by prepregnancy BMI1, Table S2: Longitudinal associations of maternal total 25(OH)D (nmol/L) and birthweight z-score, length (cm), and sum of skinfolds (mm), Table S3: Frequency of cases and controls by 25(OH)D status in the full sample and stratified by prepregnancy BMI, Figure S1: Association between 25(OH)D profiles and neonatal anthropometry.

## Abbreviations

BMI	Body Mass Index
CI	Confidence Interval
GDM	Gestational Diabetes Mellitus
GW	Gestational Week
PA	Physical Activity
SE	Standard Error
25[OH]D	Total 25-hydroxyvitamin D calculated as 25[OH]D_2_ + 25[OH]D_3_
25[OH]D_3_	25-hydroxycholecalciferol
25[OH]D_2_	25-hydroxyergocalciferol
