# Supplementary material for: Longitudinal Maternal Vitamin D Status during Pregnancy Is Associated with Neonatal Anthropometric Measures
Source: Nutrients. 2018 Nov 2;10(11):1631. doi: 10.3390/nu10111631 (PMC6265933; doi:10.3390/nu10111631)
Supplement: Supplementary file 1 [file nutrients-10-01631-s001.pdf]

Table S1. Longitudinal associations of maternal total 25(OH)D and neonatal birthweight z-score, length (cm), and sum of skinfolds stratified by prepregnancy BMI [1].

	10–14 GW			15–26 GW			23–31 GW			33-39 GW		
	Median 25(OH)D, n	β (95% CI)	P-trend[2]	Median 25(OH)D, n	β (95% CI)	P-trend[2]	Median 25(OH)D, n	β (95% CI)	P-trend[2]	Median 25(OH)D, n	β (95% CI)	P-trend[2]
Maternal 25(OH)D Status												
**Overweight/Obese BMI**												
Birthweight												
Quartile 1	47.4, 46	−0.50 (−0.86, −0.14)*		51.0, 52	−0.17 (−0.73, 0.38)		53.6, 34	−0.20 (−0.83, 0.42)		51.1, 32	−0.62 (−1.34, 0.11)	
Quartile 2	61.2, 45	−0.31 (−0.67, 0.05)		68.4, 41	−0.19 (−0.58, 0.20)		70.3, 30	0.23 (−0.52, 0.99)		69.2, 26	−0.11 (−0.68, 0.45)	
Quartile 3	75.9, 30	0.13 (−0.33, 0.59)		81.0, 29	−0.07 (−0.56, 0.41)		86.4, 33	−0.29 (−0.94, 0.35)		84.2, 26	−0.79 (−1.44, −0.14)*	
Quartile 4	91.4, 29	Reference	0.002	102.6, 26	Reference	0.52	112.9, 15	Reference	0.73	111.6, 17	Reference	0.96
Length												
Quartile 1	47.5, 40	−2.26 (−3.83, −0.69)*		50.6, 46	−0.90 (−2.51, 0.70)		54.4, 30	−2.27 (−4.51, −0.03)*		50.8, 29	−1.93 (−4.58, 0.73)	
Quartile 2	60.7, 41	−2.59 (−4.44, −0.73)*		68.4, 36	0.31 (−1.63, 2.25)		70.3, 28	−0.76 (−3.15, 1.62)		68.7, 22	0.38 (−1.82, 2.58)	
Quartile 3	75.7, 29	−0.70 (−2.40, 1.01)		81.0, 28	−0.91 (−2.75, 0.93)		86.9, 30	−0.23 (−2.76, 2.30)		84.7, 25	−2.23 (−4.72, 0.27)	
Quartile 4	90.7, 26	Reference	0.001	104.2, 25	Reference	0.76	112.9, 15	Reference	0.01	111.6, 17	Reference	0.91
Sum of Skinfolds												
Quartile 1	47.7, 38	−0.74 (−5.07, 3.60)		51.8, 43	0.77 (−3.23, 4.77)		56.2, 29	1.07 (−3.90, 6.04)		52.7, 29	−3.81 (−8.66, 1.03)	
Quartile 2	61.0, 36	−0.35 (−4.26, 3.56)		68.4, 36	3.32 (−0.25, 6.89)		70.7, 27	5.68 (−0.17, 11.53)		68.7, 21	1.80 (−2.45, 6.05)	
Quartile 3	74.7, 27	−2.13 (−5.90, 1.63)		81.0, 23	−0.84 (−4.50, 2.83)		87.4, 27	2.12 (−3.08, 7.32)		83.5, 22	−3.37 (−7.49, 0.76)	
Quartile 4	91.3, 24	Reference	0.96	104.2, 23	Reference	0.39	112.9, 15	Reference	0.74	111.4, 16	Reference	0.81
**Normal BMI**												

Birthweight												
Quartile 1	47.1, 34	0.01 (−0.44, 0.45)		51.3, 28	0.07 (−0.36, 0.51)		53.1, 13	0.43 (−0.13, 0.99)		57.5, 12	0.54 (−0.03, 1.11)	
Quartile 2	62.2, 37	−0.20 (−0.63, 0.24)		66.0, 31	−0.14 (−0.61, 0.32)		70.5, 18	0.06 (−0.6, 0.72)		69.7, 20	0.57 (−0.07, 1.20)	
Quartile 3	75.3, 38	−0.21 (−0.63, 0.21)		82.6, 45	0.28 (−0.12, 0.67)		87.7, 35	−0.18 (−0.65, 0.29)		90.8, 26	−0.07 (−0.54, 0.40)	
Quartile 4	93.1, 47	Reference	0.80	102.3, 50	Reference	0.90	112.4, 26	Reference	0.21	113.3, 28	Reference	0.03
Length												
Quartile 1	46.7, 33	0.61 (−0.86, 2.09)		51.4, 27	0.66 (−0.85, 2.16)		53.1, 13	1.70 (0.04–3.36)*		57.5, 12	2.38 (0.81, 3.95)*	
Quartile 2	62.4, 35	0.34 (−1.16, 1.84)		65.9, 29	0.03 (−1.03, 1.09)		70.5, 18	−0.23 (−1.57, 1.11)		69.7, 20	0.89 (−0.61, 2.38)	
Quartile 3	75.5, 33	−0.38 (−1.59, 0.83)		83.8, 40	0.88 (−0.48, 2.23)		87.4, 32	0.93 (−0.34, 2.20)		90.8, 24	0.80 (−0.43, 2.03)	
Quartile 4	93.2, 43	Reference	0.38	102.1, 47	Reference	0.51	112.4, 24	Reference	0.13	113.5, 25	Reference	0.001
Sum of Skinfolds												
Quartile 1	46.7, 29	−2.78 (−4.85, −0.71)*		51.6, 26	−0.94 (−3.25, 1.37)		50.1, 12	−0.42 (−3.83, 3.00)		57.5, 12	1.61 (−1.49, 4.70)	
Quartile 2	62.4, 34	−2.01 (−4.13, 0.11)		66.3, 26	−2.05 (−4.09, −0.01)*		70.6, 17	−1.82 (−4.86, 1.21)		70.4, 18	1.84 (−0.76, 4.44)	
Quartile 3	75.3, 33	−2.11 (−4.38, 0.17)		82.6, 41	−0.80 (−2.85, 1.26)		87.6, 32	−2.01 (−4.93, 0.91)		92.6, 25	0.82 (−1.57, 3.21)	
Quartile 4	93.8, 42	Reference	0.01	102.7, 44	Reference	0.16	112.9, 23	Reference	0.54	113.5, 25	Reference	0.15
**All**												
Birthweight												
Quartile 1	47.4, 80	−0.34 (−0.65, −0.02)*		51.2, 80	−0.14 (−0.47, 0.19)		53.1, 47	−0.03 (−0.38, 0.32)		53.3, 44	0.06 (−0.33, 0.46)	
Quartile 2	62.1, 82	−0.24 (−0.55, 0.08)		67.6, 72	−0.07 (−0.40, 0.26)		70.5, 48	0.17 (−0.28, 0.62)		69.6, 46	0.38 (−0.06, 0.83)	
Quartile 3	75.4, 68	−0.16 (−0.51, 0.18)		82.2, 74	0.12 (−0.22, 0.46)		87.5, 68	−0.24 (−0.65, 0.17)		86.4, 52	−0.29 (−0.68, 0.09)	
Quartile 4	92.2, 76	Reference	0.03	102.3, 76	Reference	0.29	112.9, 41	Reference	0.75	113.0, 45	Reference	0.24
Length												
Quartile 1	47.4, 73	−0.57		51.1, 73	−0.03		53.2, 43	−0.27		53.2, 41	0.59	

		(−1.79, 0.65)			(−1.11, 1.04)			(−1.56, 1.01)			(−0.68, 1.87)	
Quartile 2	62.0, 76	−0.62 (−1.89, 0.66)		66.7, 65	0.74 (−0.40, 1.89)		70.5, 46	0.37 (−0.80, 1.53)		69.1, 42	1.36 (0.03, 2.69)*	
Quartile 3	75.6, 62	−0.44 (−1.59, 0.71)		82.2, 68	0.46 (−0.75, 1.66)		87.3, 62	−0.04 (−1.47, 1.39)		86.6, 49	−0.52 (−1.74, 0.71)	
Quartile 4	92.0, 69	Reference	0.32	102.3, 72	Reference	0.81	112.9, 39	Reference	0.84	113.3, 42	Reference	0.08
Sum of Skinfolds												
Quartile 1	47.5, 67	−2.36 (−4.51, −0.22)*		51.8, 69	−1.09 (−3.16, 0.97)		54.4, 41	−1.54 (−4.09, 1.02)		54.4, 41	−1.20 (−4.02, 1.62)	
Quartile 2	61.8, 70	−1.23 (−3.18, 0.72)		67.6, 62	0.54 (−1.57, 2.64)		70.7, 44	1.32 (−1.69, 4.34)		68.7, 39	2.15 (−0.73, 5.02)	
Quartile 3	75.3, 60	−1.95 (−3.88, −0.02)*		82.2, 64	−0.83 (−2.73, 1.07)		87.6, 59	−1.55 (−4.21, 1.11)		86.6, 47	−1.70 (−4.22, 0.82)	
Quartile 4	93.2, 66	Reference	0.05	102.8, 67	Reference	0.56	112.9, 38	Reference	0.51	113.0, 41	Reference	0.91

[1] Data are presented as regression coefficients and confidence intervals (CI) and reflect the differences in neonatal anthropometry compared to the reference group (highest 25(OH)D levels); Overweight/obese body mass index (BMI) 25.0–44.9 kg/m^2, and normal BMI <25.0 kg/m^2; All models are adjusted for maternal matching characteristics (age (continuous), race, and gestational age at blood collection), and adjusted for education, insurance type, marital status, and prepregnancy BMI (continuous). [2]. *P*-trend based on robust variance estimates. *(*P*<0.005).

Table S2. Longitudinal associations of maternal total 25(OH)D (nmol/L) and birthweight z-score, length (cm), and sum of skinfolds (mm) [1].

**Maternal 25(OH)D Status**	n	10–14 GW	P	n	15–26 GW	P	n	23–31 GW	P	n	33–39 GW	P
Birthweight												
<50 nmol/L	69	−0.35 (−0.68, −0.02)	0.04	36	−0.08 (−0.51, 0.35)	0.71	17	−0.09 (−0.49, 0.31)	0.67	15	0.16 (−0.38, 0.69)	0.56
50–75 nmol/L	138	−0.22 (−0.48, 0.05)	0.11	121	−0.13 (−0.39, 0.14)	0.35	68	0.01 (−0.35, 0.36)	0.96	69	0.17 (−0.19, 0.54)	0.35
>75 nmol/L	99	Reference		145	Reference		116	Reference		103	Reference	
Length												
<50 nmol/L	50	−0.39 (−1.62, 0.84)	0.53	34	0.15 (−1.23, 1.52)	0.83	17	−0.19 (−1.72, 1.34)	0.81	14	−0.21 (−1.73, 1.32)	0.79
50–75 nmol/L	127	−0.65 (−1.56, 0.27)	0.17	108	0.37 (−0.64, 1.38)	0.47	65	−0.85 (−1.77, 0.07)	0.07	64	1.24 (0.01, 2.46)*	0.05
>75 nmol/L	103	Reference		136	Reference		108	Reference		96	Reference	
Sum of Skinfolds												
<50 nmol/L	44	−2.94 (−5.17, −0.71)*	0.01	29	−0.06 (−2.47, 2.35)	0.96	15	−2.26 (−5.25, 0.72)	0.14	12	−2.05 (−5.32, 1.23)	0.22
50–75 nmol/L	122	−1.26 (−2.86, 0.35)	0.13	105	1.12 (−0.57, 2.8)	0.20	63	0.39 (−1.94, 2.72)	0.74	63	2.28 (−0.16, 4.72)	0.07
>75 nmol/L	97	Reference		128	Reference		104	Reference		93	Reference	

[1] Data are presented as regression coefficients and confidence intervals (CI) and reflect the differences in neonatal anthropometry compared to the reference group (25(OH)D >75 nmol/L). All models are adjusted for maternal matching characteristics (age (continuous), race, and gestational age at blood collection), and adjusted for education, insurance type, marital status, and prepregnancy BMI (continuous). Models of sum of skinfolds were adjusted to account for the difference in days between birth and date of anthropometric measurement. *(*P*−value <0.005).

Table S3. Frequency of cases and controls[1] by 25(OH)D status in the full sample and stratified by prepregnancy BMI [2].

Maternal 25(OH)D Status	10–14 GW [3]	15–26 GW [3]	23–31 GW [3]	33–39 GW [3]
**Overweight/Obese BMI**				
Cases				
<50 nmol/L	16	10	7	6
50–75 nmol/L	24	24	22	20
>75 nmol/L	23	27	35	30
Controls				
<50 nmol/L	16	14	7	6
50–75 nmol/L	51	50	24	23
>75 nmol/L	22	25	17	16
**Normal BMI**				
Cases				
<50 nmol/L	7	2	0	0
50–75 nmol/L	14	12	10	13
>75 nmol/L	14	19	25	19
Controls				
<50 nmol/L	17	10	6	3
50–75 nmol/L	51	36	12	13
>75 nmol/L	54	76	40	39
**All**				
Cases				
<50 nmol/L	23	12	7	6
50–75 nmol/L	38	36	32	33
>75 nmol/L	37	46	60	49
Controls				
<50 nmol/L	33	24	13	9
50–75 nmol/L	102	86	36	36
>75 nmol/L	76	101	57	55

[1] Cases represent women with a diagnosis of Gestational Diabetes Mellitus (GDM); controls represent women without GDM who were matched to cases based on age, race, and gestational week at each blood collection visit. [2] Overweight/obese body mass index (BMI) 25.0–44.9 kg/m^2, and normal <25.0 kg/m^2. [3] Gestational Week (GW).

Figure S1. Association between 25(OH)D profiles [1] and neonatal anthropometry [2].

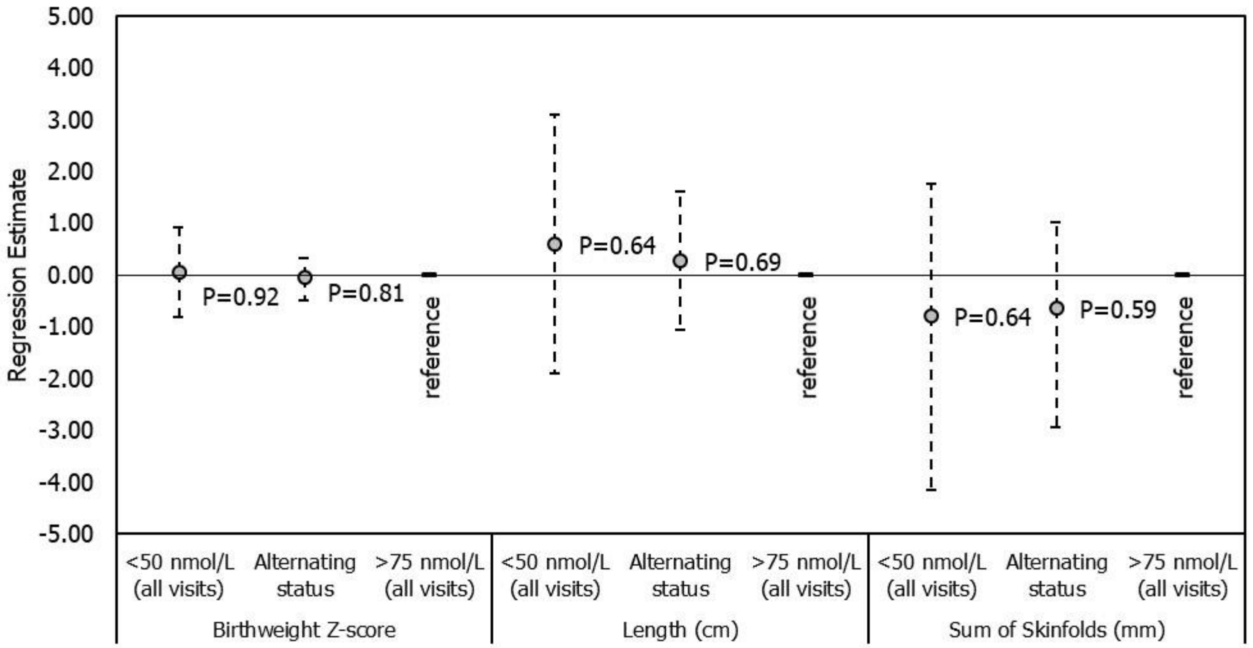

Figure 1. We examined whether the change in maternal 25(OH)D status across pregnancy was associated with neonatal anthropometric measure for women who had 25(OH)D at all four time-points (*N* = 214). [1] We grouped women into three profiles based on their 25(OH)D concentrations measured 4-times across pregnancy as 1) consistently < 50 nmol/L (*n* = 10), 2) an alternating status ranging across all concentration categories (*n* = 153), and 3) consistently >75 nmol/L (*n* = 51).
[2] All models are adjusted for maternal matching characteristics (age (continuous), race, and gestational age at from 1st blood collection to last blood collection), and adjusted for education, insurance type, marital status, and prepregnancy BMI (continuous). Models of sum of skinfolds were adjusted to account for the difference in days between birth and date of anthropometric measurement.
